# Rapid wide QRS tachycardia with an unknown cause

**DOI:** 10.1111/anec.12959

**Published:** 2022-04-16

**Authors:** Dalong Hu, Jingxiu Li

**Affiliations:** ^1^ Department of Electrocardiography Dancheng County Central Hospital Zhoukou China; ^2^ Department of Electrocardiography, The First Affiliated Hospital of USTC, Division of Life Sciences and Medicine University of Science and Technology of China Hefei China

**Keywords:** 1:1 atrioventricular conduction, atrial flutter, Wolff–Parkinson–White syndrome

## Abstract

One‐to‐one atrioventricular conduction during atrial flutter is one of the most severe life‐threatening arrhythmias and is hemodynamically perilous. Rapid wide QRS tachycardia often not only occurs in patients with ventricular tachycardia but is also found in supraventricular tachycardia/atrial flutter with preexistent QRS prolongation, supraventricular tachycardia/atrial flutter with QRS prolongation caused by an IC antiarrhythmic drug, and supraventricular tachycardia/atrial flutter with preexcitation. Furthermore, atrial flutter with 1:1 AVC via an accessory pathway is an uncommon presentation of Wolff‐Parkinson–White syndrome. We present a case of atrial flutter with 1:1 rapid AVC in the presence of Wolff–Parkinson–White syndrome. Physicians should be familiar with the rapid wide QRS complex ECG pattern associated with AFL with 1:1 AVC via an accessory pathway. Establishing the definitive diagnosis is essential for selecting an appropriate treatment strategy for improving outcomes.

## CASE

**FIGURE 1:**
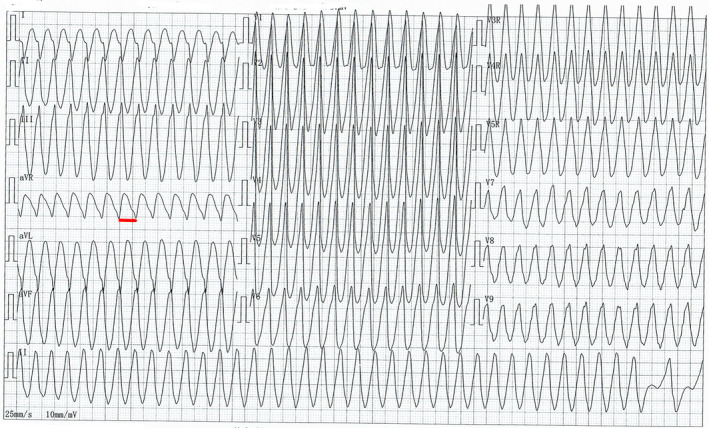
ECG was performed upon admission to the emergency department

**FIGURE 2:**
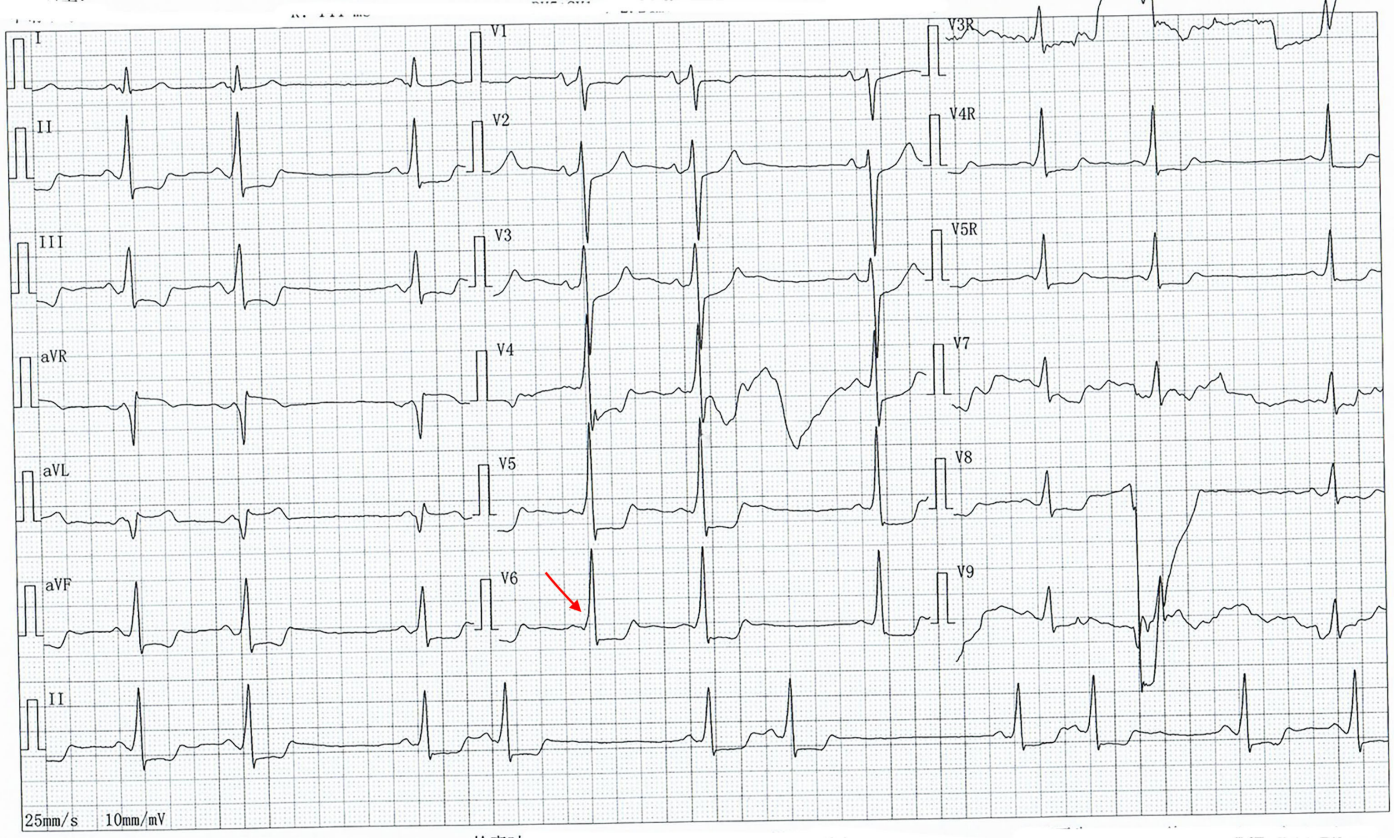
ECG after cardioversion displaying sinus rhythm with ventricular preexcitation and frequent premature atrial contractions

**FIGURE 3:**
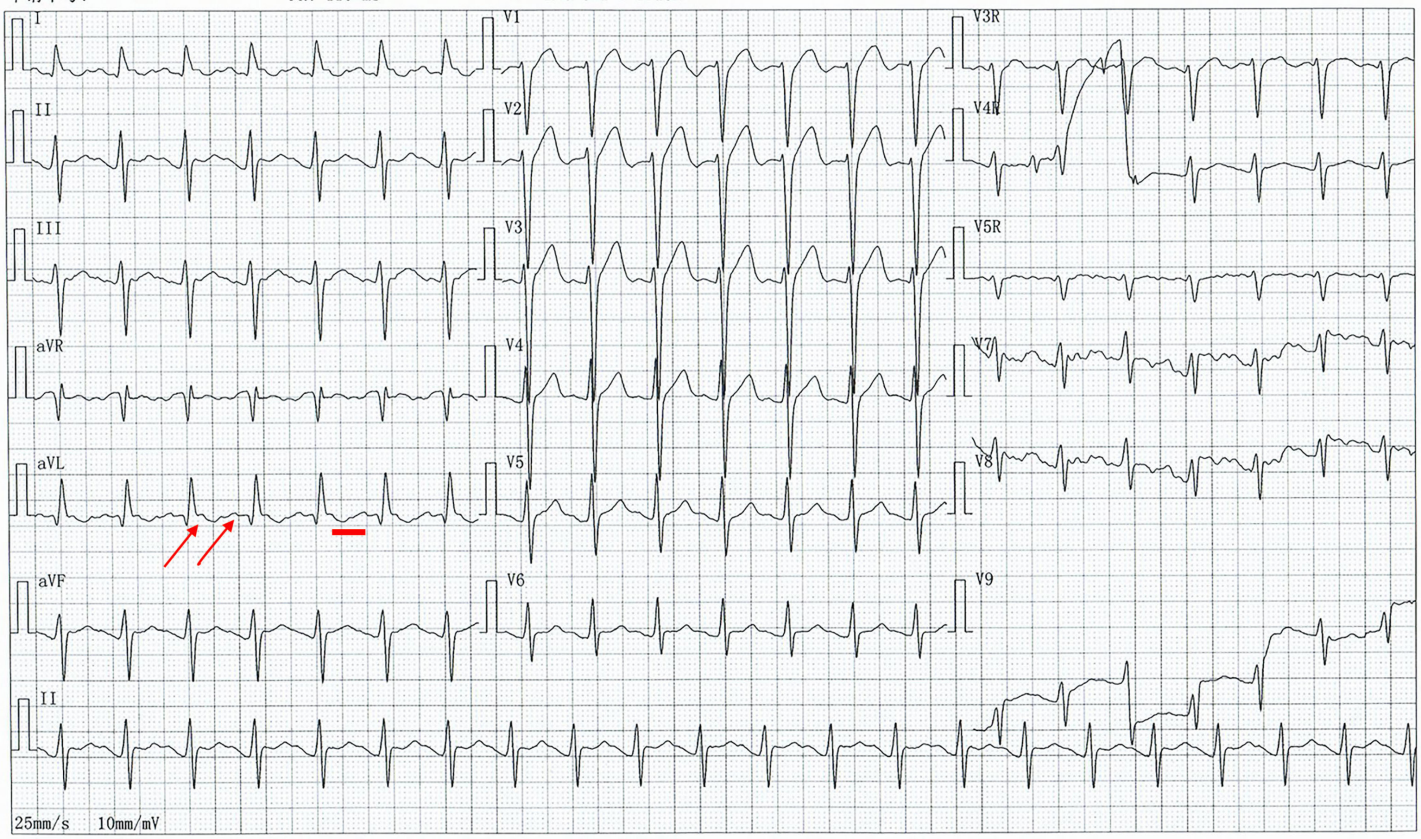
ECG showing atrial flutter with 2:1 atrioventricular conduction. The rate of the atrial flutter wave is identical to the rate of the initial tachyarrhythmia

A 52‐year‐old male patient complaining of palpitations and chest discomfort for the past 90 min presented to our emergency department. The patient's history was significant for a coronary intervention one year ago. His blood pressure was 81/54 mmHg, and his heart rate was 240 bpm. An urgent ECG was performed and is shown in Figure 1. Electrical cardioversion and pharmacological therapy were administered under the guidance of a cardiovascular physician. Laboratory test results were all within normal limits. The ECG revealed a regular wide complex tachycardia at 240 beats per minute. The possible differential diagnoses include ventricular tachycardia, supraventricular tachycardia/atrial flutter with preexistent QRS prolongation, supraventricular tachycardia/atrial flutter with QRS prolongation caused by an IC antiarrhythmic drug, and supraventricular tachycardia/atrial flutter with preexcitation. Several features suggesting ventricular tachycardia can be identified: In V2, the interval between the beginning of the R wave to the nadir of the S wave is more than 100 ms. The RS/QRS ratio in lead V6 is 0.61 (cutoff 0.41). The initial R wave in V1, initial R wave >40 ms in V2, and R wave peak time > 50 ms in lead II produce a ventricular tachycardia score of 3. The cardiovascular physician treated the patient with electrical cardioversion and amiodarone. A second ECG, obtained after cardioversion, showed a sinus rhythm with ventricular preexcitation and frequent premature atrial contractions (Figure 2). Three hours later, the ECG showed atrial flutter (AFL) with 2:1 atrioventricular conduction (AVC) (Figure 3). The rate of the atrial flutter wave was identical to the rate of the initial tachyarrhythmia. Retrospectively, the initial tachyarrhythmia was diagnosed as AFL with 1:1 atrioventricular conduction via an accessory pathway.

## DISCUSSION

One‐to‐one atrioventricular conduction during AFL is one of the most severe life‐threatening arrhythmias and is hemodynamically perilous.(Hegde & Kumar, 2016) It has been reported in patients with AFL who received sodium channel blockers, such as flecainide, quinidine, procainamide, and disopyramide.(Brembilla‐Perrot et al., 2001) These drugs slowed both the atrial conduction and the AFL rate itself. However, Class I antiarrhythmic drugs are less likely to be used in modern clinical treatment to convert AFL to sinus rhythm. Therefore, drug‐induced AFL with 1:1 AVC, which was once common, is becoming rare. AFL with 1:1 atrial ventricular conduction might also be triggered by sympathetic stimulation, or may occur spontaneously.(Turitto et al., 2009) Previously, our team reported AFL with rapid, wide QRS 1:1 AVC in a patient following surgical repair of an atrial septal defect and tricuspid valvuloplasty.

AFL with 1:1 AVC via an accessory pathway is an uncommon presentation of Wolff–Parkinson–White syndrome. In 2014, Jessie reported a case of confirmed AFL with 1:1 AVC and revealed an accessory pathway consistent with Wolff–Parkinson–White syndrome, despite a lack of ECG findings of preexcitation during sinus rhythm. In our report, the patient showed a preexcitation accessory pathway during sinus rhythm. During his hospitalization, we also recorded AFL with 2:1 AVC and atrial fibrillation in the presence of Wolff–Parkinson–White syndrome. The rate of the AFL wave was identical to that of the initial tachyarrhythmia. Retrospectively, this tachyarrhythmia was diagnosed as AFL with 1:1 rapid AVC in the presence of Wolff–Parkinson‐White syndrome.

In conclusion, AFL with rapid 1:1 AVC is an uncommon but challenging and potentially fatal arrhythmia, especially in patients with Wolff–Parkinson–White syndrome. AFL with 1:1 rapid AVC in Wolff–Parkinson–White syndrome must be differentiated from ventricular tachycardia, supraventricular tachycardia with aberrant conduction, supraventricular tachycardia with bundle branch block, and supraventricular tachycardia with intraventricular block. Firstly, it is important to take into account any underlying heart disease. Secondly, obtaining an ECG before and after pharmacological therapy is beneficial for accurate diagnosis. It is crucial to be conscious of the differential diagnosis of AFL with rapid, wide QRS 1:1 AVC to avoid misdiagnoses and mismanagement.

## CONFLICT OF INTEREST

Founders did not play any role in this study design, data collection, analysis, the decision to publish, or preparation of the manuscript.

## AUTHOR CONTRIBUTION

Dalong Hu contributed significantly to data collection and manuscript preparation. Jingxiu Li performed the analysis with discussion. All the authors agree on the order in which their names will be listed in the manuscript.

## ETHICAL APPROVAL

We identify that the ethics committee of Dancheng County Central Hospital has approved the case and that this case conforms to recognized standards, the Declaration of Helsinki.

## Data Availability

Data sharing does not apply to this article as no datasets were generated or analyzed during the current study.
